# A Link between Mitochondrial Dysregulation and Idiopathic Autism Spectrum Disorder (ASD): Alterations in Mitochondrial Respiratory Capacity and Membrane Potential

**DOI:** 10.3390/cimb43030157

**Published:** 2021-12-16

**Authors:** Hazirah Hassan, Fazaine Zakaria, Suzana Makpol, Norwahidah Abdul Karim

**Affiliations:** Department of Biochemistry, Faculty of Medicine, Universiti Kebangsaan Malaysia, Kuala Lumpur 56000, Malaysia; p90607@siswa.ukm.edu.my (H.H.); fazaine@yahoo.com.my (F.Z.); suzanamakpol@ppukm.ukm.edu.my (S.M.)

**Keywords:** autism, mitochondria, mitochondrial respiration, high-resolution respirometry, oxidative phosphorylation, mitochondrial membrane potential

## Abstract

Autism spectrum disorder (ASD) is a neurological disorder triggered by various factors through complex mechanisms. Research has been done to elucidate the potential etiologic mechanisms in ASD, but no single cause has been confirmed. The involvement of oxidative stress is correlated with ASD and possibly affects mitochondrial function. This study aimed to elucidate the link between mitochondrial dysregulation and idiopathic ASD by focusing on mitochondrial respiratory capacity and membrane potential. Our findings showed that mitochondrial function in the energy metabolism pathway was significantly dysregulated in a lymphoblastoid cell line (LCL) derived from an autistic child (ALCL). Respiratory capacities of oxidative phosphorylation (OXPHOS), electron transfer of the Complex I and Complex II linked pathways, membrane potential, and Complex IV activity of the ALCL were analyzed and compared with control cell lines derived from a developmentally normal non-autistic sibling (NALCL). All experiments were performed using high-resolution respirometry. Respiratory capacities of OXPHOS, electron transfer of the Complex I- and Complex II-linked pathways, and Complex IV activity of the ALCL were significantly higher compared to healthy controls. Mitochondrial membrane potential was also significantly higher, measured in the Complex II-linked pathway during LEAK respiration and OXPHOS. These results indicate the abnormalities in mitochondrial respiratory control linking mitochondrial function with autism. Correlating mitochondrial dysfunction and autism is important for a better understanding of ASD pathogenesis in order to produce effective interventions.

## 1. Introduction

Autism spectrum disorder (ASD) is a heterogeneous neurodevelopmental disorder characterized by a combination of impairments in social communication and interaction, sensory anomalies, repetitive behaviors, and varying levels of intellectual disability. The global prevalence of ASD has increased dramatically 20- to 30-fold over the past decades [1] and the World Health Organization estimated that 1 in 160 children is affected by ASD [2]. The recent increase in ASD underscores the importance of expanding research into risk factors and effective interventions. Whilst most studies focus on effective interventions on promoting communication, social skills, and education [3], the underlying cause of idiopathic autism remains unclear. Up to the present, no significant underlying cause and no pharmacological medication is currently established as a treatment. Tracking the increase in ASD prevalence poses unique challenges because of its complex nature, lack of diagnostic biomarkers, and challenging diagnostic criteria.

Over the last decade, increasing attention has been paid to the mitochondrial physiology that may underlie some of the symptoms of ASD. Often mitochondrial dysfunction (mtD) is associated with the inability of mitochondria to transform the chemical energy in the form of ATP. mtD manifests as a change in mitochondrial structure, membrane potential, mitochondrial enzyme activity, and dysregulated mitochondrial energy metabolism, which is frequently linked to oxidative/nitrosative stress [4]. Reports showed that autism was correlated with increased oxidative stress that might subsequently trigger oxidative damages, which could be involved in the pathogenesis of the disorder [5]. mtD is also implicated with other neurodegenerative diseases, such as Alzheimer’s disease, Huntington’s disease and Parkinson’s disease [6,7,8,9], and aging [10]. However, the factors that cause mtD are still unknown. Some studies suggest that mtD is a consequence of dysreactive immunity and altered calcium signaling [11], malnutrition and vitamin deficiencies, exposure to environmental toxicants, and oxidative stress [12,13,14].

At cellular and molecular levels, mtD results in many negative downstream consequences. These include reduced synaptic neurotransmitter release in neurons that have high firing rates, such as inhibitory γ-aminobutyric acid interneurons, which in turn could result in reduced inhibition of neurotransmitter release [15] and the relative increase in the excitatory-to-inhibitory ratio observed in ASD patients [16]. Mitochondria are concentrated in the dendritic and axonal termini where they play an important role in ATP production, calcium homeostasis, and synaptic plasticity [4], pointing toward a connection between mtD and ASD. Importantly, brain cells have a high aerobic energy demand. Thus, a dysregulation of pathways in mitochondrial energy metabolism (i.e., oxidative phosphorylation (OXPHOS) and ATP turnover) might be the underlying cause of ASD pathogenesis.

Biochemical diagnosis of mtD is frequently restricted to the measurement of lactate, pyruvate, and some amino acids in plasma, cerebrospinal fluid, and urine [4,17], whereas recent studies reveal the linkage between the electron transport chain and mtD [18]. Nevertheless, in our study, the mitochondrial function of lymphoblastoid cell lines (LCL) derived from an autistic child (ALCL) was investigated by the determination of cytochrome *c* oxidase (CIV) activity, respiration of intact and permeabilized cells, and mitochondrial membrane potential (mtMP).

The mitochondrial function studied here involves electron transfer in an NADH-linked pathway (N-pathway), which is obtained by the addition of NADH-generating substrate combinations of pyruvate (P), glutamate (G), and malate (M). This is linked to Complex I (CI). The electron transfer in a succinate-linked pathway (S-pathway) is obtained by the addition of succinate (S), which is the only substrate of Complex II (CII). Electron transfer from both pathways combines (NS-pathway) and converges at the Q-junction. The downstream electron flow is catalyzed by Complex III (CIII) and CIV. In the presence of fuel substrates and ADP, the OXPHOS capacity (*P*) can be measured. The electron capacity (ET capacity, *E*) is obtained at optimum uncoupler concentrations, such as CCCP. On the other hand, the measurement of mtMP is performed using a lipophilic cation, which accumulates in the negatively charged mitochondrial matrix [19,20].

This is the first report on the elucidation of mitochondrial functionality in ALCL using high-resolution respirometry.

## 2. Materials and Methods

### 2.1. Chemicals

All chemicals were purchased from Sigma-Aldrich (Sigma-Aldrich, Burlington, MA, USA) unless otherwise stated.

### 2.2. Cell Culture

Both lymphoblastoid cell lines (LCLs) were purchased from Autism Genetic Resource Exchange (AGRE; Los Angeles, CA, USA). The cells are derived from male siblings in which one sibling has been diagnosed with ASD (LCL derived from an autistic child, ALCL; Cell ID: 065604, aged 11) and the healthy control was from an apparently healthy sibling with no observation of behavioral and neurological disorders (LCL derived from a non-autistic sibling, NALCL; Cell ID: 065603, aged 12). The parents of the siblings were healthy and the ethnicity of the family was classified as non-Hispanic or Latino. The DNA of the ASD patient was tested for mutation of the Fragile-X syndrome gene, the *FMR1* gene, and no known mutation was found.

The cell lines were cultured in complete culture media that consist of Rosewell Park Memorial Institute (RPMI) 1640 media supplemented with a volume fraction of 0.15% fetal bovine serum, 2 mM l-glutamine, 100 U∙mL^−1^ penicillin, and 100 U∙mL^−1^ streptomycin (Gibco, Carlsbad, CA, USA) at 37 °C in an incubator with 5% CO_2_. The LCLs used in this study were within passages 8 to 10.

LCLs were centrifuged at 130× *g* and 5 × 10^6^ cells were resuspended in 300 µL respiration medium, MiR05 (0.5 mM EGTA, 3 mM MgCl_2_, 60 mM lactobionic acid, 20 mM taurine, 10 mM KH_2_PO_4_, 20 mM HEPES, 110 mM D-sucrose, 1.0% (*w*/*v*) bovine serum albumin) and pH was adjusted to 7.1 with KOH at 30 °C.

### 2.3. High–Resolution Respirometry

All experiments were performed using a modular instrument for high-resolution respirometry (HRR) and fluorometry, the Oxygraph-2k (O2k; Oroboros Instruments, Innsbruck, Austria). The temperature of the experimental chamber was kept at 37 °C with constant stirring at 750 rpm to ensure a homogeneous oxygen distribution in the chamber without cell disruption. Air calibration of the oxygen sensors was performed daily. DatLab 7 software (Oroboros Instruments) was used for real-time data acquisition and analysis. The oxygen concentration was kept at and below air saturation (normoxic conditions). The O2k chambers were filled with 2.0 mL MiR05 equilibrated at 37 °C. Titrations were performed manually by injection into O2k chambers using precalibrated Hamilton microsyringes. Substrate concentrations and respiratory states are listed in Table 1 and Table 2, respectively. Oxygen consumption rates were normalized to cell number and expressed as O_2_ flow per cell [amol∙s^−1^∙cell^−1^].

### 2.4. Cytochrome c Oxidase (CIV) Activity Assay

**Figure 1:**
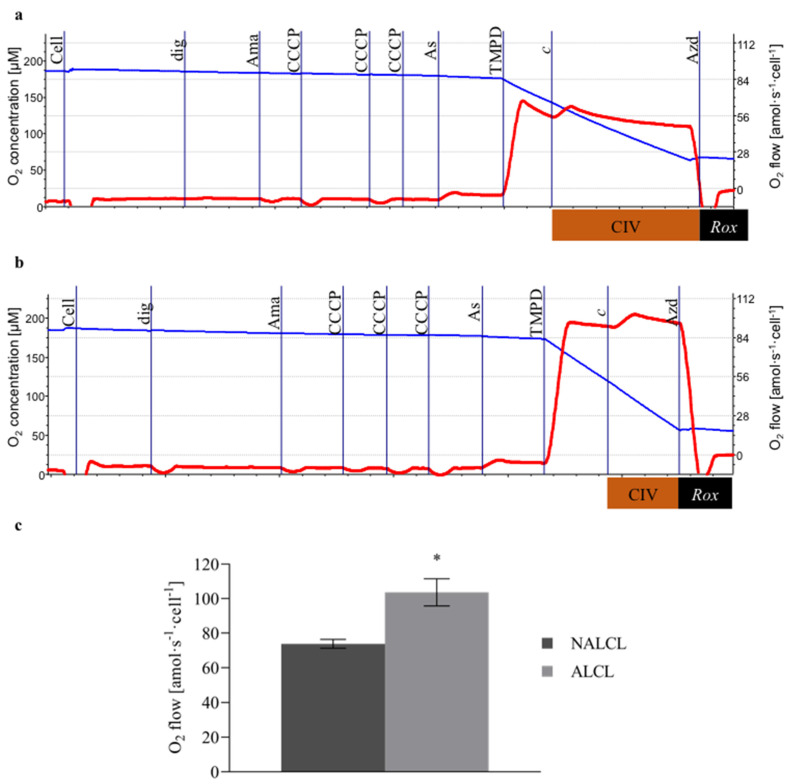
Cytochrome *c* oxidase (CIV) activities of NALCL and ALCL. (**a**,**b**) are the protocols for measuring CIV activities in NALCL and ALCL, respectively. Titrations: Cell, dig (digitonin, cell permeabilization), Ama (antimycin A, CIII inhibition), CCCP (oxidative phosphorylation uncoupling), As (ascorbate, maintaining TMPD in a reduced state), TMPD (reducing cytochrome *c*), *c* (cytochrome *c* integrity of outer mt-membrane), and Azd (azide, CIV inhibition). Blue plots indicate O_2_ concentration and red plots are the O_2_ consumption expressed per cell. (**c**) The total O_2_ consumption rate was baseline-corrected for autoxidation after inhibition of CIV. Results are expressed as means ± S.D. * denotes *p* < 0.05 compared to NALCL as determined by *t*-test.

Cells in MiR05 were titrated into each chamber, followed by titration of digitonin (7.5 µg∙mL^−1^ for NALCL, 27.5 µg∙mL^−1^ for ALCL). CIII was inhibited by the addition of antimycin A (2.5 µM). Stepwise titration of the protonophore CCCP (0.5 μM steps) leads to proton leakage across the mitochondrial inner membrane. Ascorbate (2 mM) was added before the addition of TMPD (0.5 mM) to avoid uncontrolled autoxidation and to maintain TMPD in a reduced state. The artificial substrate TMPD reduced cytochrome *c*. The activity of CIV was later inhibited by the addition of sodium azide (100 mM). Chemical background due to the autoxidation of ascorbate, TMPD, and cytochrome *c* was assessed after the inhibition of CIV by sodium azide. Chemical background correction was performed using DatLab 7. The CIV activity was expressed as a function of oxygen concentration. The protocols for measuring CIV activities in NALCL and ALCL were shown in Figure 1a and Figure 1b respectively.

### 2.5. Respiration

**Figure 2:**
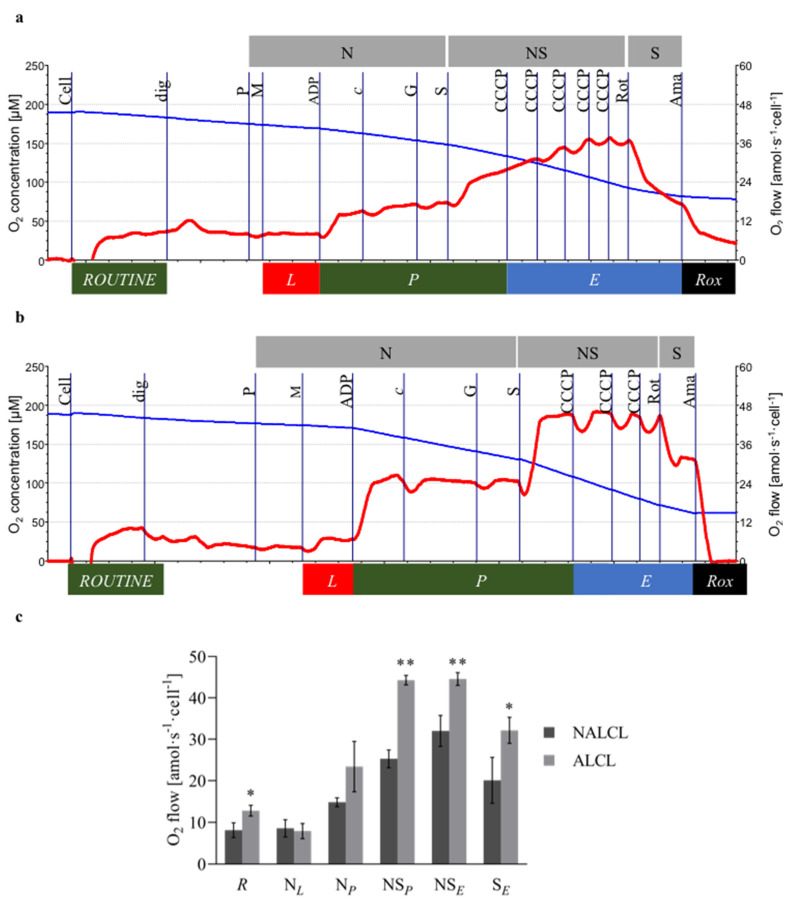
Mitochondrial respiration of NALCL and ALCL. (**a**,**b**) are the protocols for measuring respiration in NALCL and ALCL, respectively. Titrations: Cell, dig (digitonin, cell permeabilization), P and M (pyruvate and malate, non-phosphorylating N-LEAK respiration, N(GM)*_L_*_(n)_), ADP (N-OXPHOS capacity, N(GM)*_P_*), *c* (cytochrome *c* integrity of outer mt-membrane), G (glutamate, N-OXPHOS capacity, N(PGM)*_P_*), S (succinate, NS-OXPHOS, NS*_P_*), CCCP (NS-ET capacity, NS*_E_*), Rot (rotenone, CI inhibition, S-ET capacity, S*_E_*), and Ama (antimycin A, CIII inhibition, *Rox*). Oxygen consumption was corrected for *Rox*. Blue plots indicate O_2_ concentration and red plots are the O_2_ consumption expressed per cell. (**c**) The *Rox*-corrected respiration: ROUTINE respiration, *R*, was measured in non-permeabilized cells in MiR05. After plasma membrane permeabilization, five respiratory states were sequentially established to measure NADH-linked LEAK respiration with pyruvate and malate, N*_L_*, OXPHOS capacity, N*_P_*, NS-pathway OXPHOS capacity, NS*_P_*, and NS- and S-pathway ET capacity, NS*_E_* and S*_E_*, where S indicates the succinate pathway. Results are expressed as means ± S.D. * denotes *p* < 0.05 while ** denotes *p* < 0.01 compared to NALCL as determined by *t*-test.

The addition of the NADH-linked substrates pyruvate (P; 5 mM) and malate (M; 2 mM) induced non-phosphorylating LEAK respiration, N(GM)*_L_*. Subsequently, N-OXPHOS capacity, N(GM)*_P_*, was measured after the addition of a saturating concentration of ADP (2.5 mM). Cytochrome *c* (10 µM) was added to test the integrity of the mitochondrial outer membrane damage. Glutamate (10 mM) was added to stimulate multiple NADH hydrogenases, N(PGM)*_P_*. The addition of succinate (S; 10 mM) stimulated the OXPHOS capacity of the combined N- and S-pathways (NS*_P_*). Stepwise titration of the CCCP (0.5 μM steps) was used to measure the capacity of the ETS (NS*_E_*). Then, CI was inhibited by rotenone (0.5 µM) to measure S-ET capacity (S*_E_*). The inhibition of CIII by antimycin A (2.5 µM) provided a measure of *Rox*. For further methodological details see [21]. The protocols for measuring respiration in NALCL and ALCL were shown in Figure 2a and Figure 2b respectively.

### 2.6. Mitochondrial Membrane Potential

**Figure 3:**
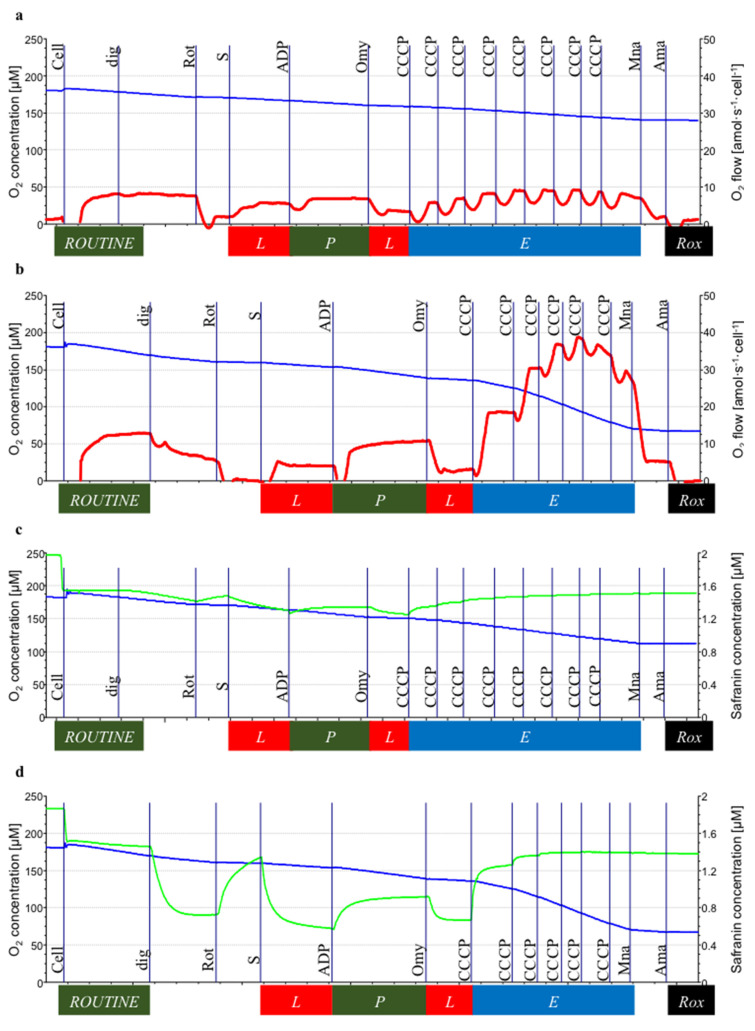

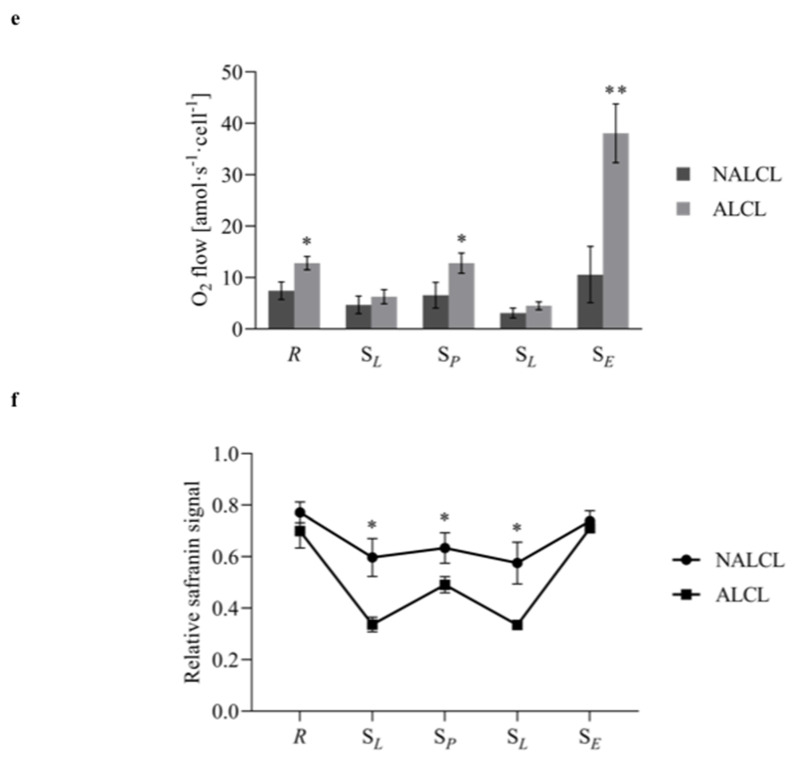
Succinate-linked respiration of NALCL and ALCL measured simultaneously with mitochondrial membrane potential in the presence of safranin. (**a**,**b**) are the protocols for measuring respiration in NALCL and ALCL, respectively, while (**c**,**d**) are the protocols for measuring mitochondrial membrane potential, mtMP using safranin concentration in NALCL and ALCL, respectively. Before the cells were added, safranin was calibrated up to 2 µM. The initial drop to 1.6 µM safranin is mainly due to unspecific binding upon the addition of cells. Low safranin concentrations indicate high mtMP. Titrations: Cell, dig (digitonin, cell permeabilization), Rot (rotenone, CI inhibition), S (succinate, S-LEAK respiration, S*_L_*_(n)_), ADP (S-OXPHOS capacity, S*_P_*), Omy (oligomycin, S-LEAK, S*_L_*_(Omy)_), CCCP (S-ET capacity, S*_E_*), Mna (malonate, CII inhibition), and Ama (antimycin A, CIII inhibition, *Rox*). Oxygen consumption was corrected for *Rox*. Blue plots indicate O_2_ concentration, red plots are the O_2_ consumption expressed per cell, and neon plots indicate safranin concentrations. (**e**) The *Rox*-corrected respiration and (**f**) the relative safranin signal: ROUTINE state, *R*, and in four respiratory states in permeabilized cells: S-linked LEAK respiration in the absence of adenylates, S*_L_*_(n)_, S-linked OXPHOS capacity, S*_P_*, S-linked LEAK respiration after inhibition of ATP synthase by oligomycin, S*_L_*_(Omy)_, and S-linked ET capacity, S*_E_*. Results are expressed as mean ± S.D. * denotes *p* < 0.05 while ** denotes *p* < 0.01 compared to NALCL as determined by Student’s *t*-test.

The Fluorescence-Sensor Blue of the O2k-Fluorescence LED2-Module was used with filter sets for safranin. Two sensors were inserted through the front windows of the O2k-chambers. Following air calibration, the chamber illumination was switched off. HRR provides simultaneous measurement of respiration and the safranin signal in each chamber [20]. The polarization voltage regulating light intensity was set at 500 mV at gain 1000. Safranin is a fluorophore with an excitation wavelength of 495 nm and an emission wavelength of 587 nm, and it is a lipophilic cation that accumulates in mitochondria depending on the inside negative potential in energized mitochondria [19,20]. Upon accumulation in the matrix, safranin undergoes a change in absorption and self-quenching of fluorescence [25,26]. Fluorescence intensity is linearly related to mtMP [27]. For calibration, a 200 µM stock solution of safranin was titrated in five steps to obtain final concentrations of 0.25, 0.5, 1.0, 1.5, and 2.0 µM, obtaining a linear increase of the fluorescence signal as a function of safranin concentration in the chamber. Cells were then added to the chambers. In the absence of respiratory inhibitors, the mtMP builds up on the basis of endogenous substrates, and a corresponding amount of the dye accumulates in the mitochondrial matrix (i.e., the initial decline of the fluorescence signal). In addition, a fraction of safranin binds non-specifically to cell membranes. Therefore, the free safranin concentration is lower than the total safranin concentration added to the chamber [20]. As safranin inhibits N-pathway OXPHOS capacity [19,20], only S-linked respiration was measured. S*_L_*_(n)_ was induced by adding succinate (10 mM) in the presence of rotenone (0.5 µM) that inhibited CI. The titration of rotenone prevents the accumulation of oxaloacetate, which is a potent inhibitor of CII. Subsequently, S*_P_* was measured after the addition of a saturating concentration of ADP (2.5 mM). S*_L_*_(Omy)_ was induced by adding oligomycin (2.5 µM) that inhibited F_1_F_0_-ATPase (ATP synthase). Stepwise titration of the CCCP (0.5 μM steps) was used to measure S*_E_*. The inhibition of CIII by antimycin A (2.5 µM) provided a measure of *Rox*. Figure 3c,d were the protocols for measuring mitochondrial membrane potential (mtMP) using safranin concentration in NALCL and ALCL, respectively. The *Rox*-corrected respiration was shown in Figure 3e.

### 2.7. Data Analysis

Oroboros DatLab 7 was used to calculate respiration corrected for instrumental background oxygen flux and graphical presentation of experimental data. Three variants of cell lines for ALCL and NALCL were used and three independent experiments were performed for each protocol type. Data are expressed as means ± standard deviation. Statistical analyses using SPSS statistical software version 16 and *t*-test were applied to determine the significant differences among the groups, where *p* < 0.05 was considered significant.

## 3. Results

### 3.1. Complex IV Activity

The CIV activity of NALCL was 55.9 ± 10.4 amol∙s^−1^∙cell^−1^, while the activity in ALCL was 1.73-fold higher (96.7 ± 15.9 amol∙s^−1^∙cell^−1^) (Figure 1c).

### 3.2. Mitochondrial Respiration

In this study, the first respiration rate measured in both cell lines is the ROUTINE respiration which represents energy demand under steady-state conditions. ROUTINE respiration, in ALCL, was significantly higher (12.8 ± 1.3 amol∙s^−1^∙cell^−1^) compared to NALCL (8.1 ± 1.8 amol∙s^−1^∙cell^−1^). A digitonin concentration, Dig, of 7.5 µg∙mL^−1^ was optimal for NALCL and 27.5 µg∙mL^−1^ for ALCL for complete permeabilization of the plasma membrane without affecting the mitochondrial membranes.

N-LEAK respiration with pyruvate and malate, N*_L_*, was 8.6 ± 2.1 and 7.9 ± 1.8 amol∙s^−1^∙cell^−1^ in NALCL and ALCL, respectively (Figure 2c). After the addition of ADP, the N-OXPHOS capacity, N*_P_*, in NALCL and ALCL was 14.8 ± 1.1 and 23.4 ± 6.1 amol∙s^−1^∙cell^−1^, respectively (Figure 2c). This yields OXPHOS coupling efficiencies, (*P − L*)/*P*, of 0.42 and 0.66 for NALCL and ALCL, respectively.

Further stimulation of respiration with succinate activates convergent entry through the S-linked pathway via CII in addition to the N-pathway through CI, which results in NS-linked OXPHOS capacity (NS*_P_*). The NS*_P_* of NALCL increased 1.7-fold to 25.3 ± 2.1 amol∙s^−1^∙cell^−1^, indicating a strongly additive effect. The NS*_P_* of ALCL was significantly higher than in NALCL (44.3 ± 1.2 amol∙s^−1^∙cell^−1^), which is a 1.9-fold increase with respect to N_P_.

The non-coupled NS-ET capacity, NS*_E_*, of ALCL, was 44.6 ± 1.5 amol∙s^−1^∙cell^−1^, which was significantly higher than in NALCL (32.0 ± 3.8 amol∙s^−1^∙cell^−1^).

After inhibition of the N-pathway by rotenone, S-ET capacity (S*_E_*) was 20.1 ± 5.5 and 32.2 ± 3.2 amol∙s^−1^∙cell^−1^ in NALCL and ALCL, respectively. The NS-linked *P/E* ratio (NS_*P/E*_) in NALCL (0.21) was higher than in ALCL (0.01). The *P/E* coupling control ratio describes the function of the phosphorylation system as a crucial controller limiting OXPHOS capacity. A ratio of 1.0 indicates no limitation by the phosphorylation system.

### 3.3. Mitochondrial Membrane Potential

MtMP was measured in the S-pathway simultaneously with respiration in the same chamber. Rotenone and succinate were added simultaneously to establish the LEAK state in the absence of exogenous ADP and ATP (no adenylates), S*_L_*_(*n*)_. S*_L_* was similar in NALCL and ALCL (4.7 ± 1.7 and 6.3 ± 1.4 amol∙s^−1^∙cell^−1^, respectively). OXPHOS capacity, S*_P_* was significantly lower in NALCL than ALCL (6.6 ± 2.5 and 12.8 ± 2.0 amol∙s^−1^∙cell^−1^, respectively). In both cell lines, the inhibition of ATPase by oligomycin inhibited LEAK respiration, S*_L_*_(Omy)_, was below the level of S*_L_*_(*n*)_, indicating an overestimation of LEAK in the absence of inhibition of the phosphorylation system due to some recycling of endogenous ATP to ADP by ATPases (Figure 3a,b). OXPHOS coupling efficiencies were 0.29 and 0.51 in the absence of oligomycin, but 0.53 and 0.65 in the presence of oligomycin for NALCL and ALCL, respectively. The S-pathway ET capacity in the mtMP protocol was obtained by stepwise titration of CCCP up to reaching maximum respiration.

In the S-pathway with electron entry into the Q-junction through CII and only two coupling sites downstream, the *P/E* ratios increased to 0.67 and 0.60 in NALCL and ALCL, respectively, indicating the lower limit in the phosphorylation system. On the other hand, the LEAK/OXPHOS (S*_L_*_/*P*_) ratio depicts respiration triggered by membrane leakiness in comparison to OXPHOS capacity. The mitochondrial membrane leakiness appeared higher in NALCL with an *L/P* ratio of 0.72, while in ALCL, the *L/P* ratio was 0.49.

During ROUTINE respiration of intact cells, the safranin signals showed no significant difference between NALCL and ALCL (Figure 3f). After plasma membrane permeabilization, the mtMP increased in both cell lines as S-linked respiration was stimulated by the addition of succinate in the presence of Rot, as reflected by a decline of safranin fluorescence. The mtMP in ALCL showed a higher increase than in NALCL during LEAK (*p* < 0.01) and OXPHOS (*p* < 0.05; Figure 3f). There was no significant difference between mtMP in NALCL and ALCL during ET capacity measurement.

## 4. Discussion

mtD is often linked with oxidative stress as damaged mitochondria not only produce more reactive oxygen species (ROS, including H_2_O_2_) and reactive nitrogen species through OXPHOS, but mitochondria are also becoming more vulnerable to oxidative stress [28]. In the present study, we investigated the mtD concerning CIV activity, mitochondrial respiration through N-pathway (CI-linked), S-pathway (CII-linked), and both pathways as combined NS-pathway (CI- and CII-linked), and mtMP as the key markers of mitochondrial function.

The first respiratory state measured in this study is ROUTINE respiration. ROUTINE respiration is the respiration controlled by intrinsic energy demand. It represents energy demand under steady-state conditions. During ROUTINE respiration, cell respiration relies on endogenous substrates in the cells only.

The higher ROUTINE respiration rate in ALCL as compared to in NALCL showed that the intrinsic energy demand under steady-state conditions in ALCL is higher as compared to in NALCL. This situation is considered to be a compensatory response to the increased intrinsic energy demand in ALCL. Ideally, bioenergetic efficiency is achieved when there is a balance between energy supply and energy demand [29]. In response to changes in energy demand, mitochondria respond by adjusting both their capacity and efficiency of ATP production [29]. This might be reflected by the higher OXPHOS capacity in ALCL as compared to in NALCL. In an intact cell with high energy demand, nutrient utilization and its availability have very significant control over respiration as compared to ATP turnover [29].

It has been proposed that the increased respiration rate leads to higher oxidative stress and alterations in mitochondrial energy metabolism [30]. The higher OXPHOS capacity in ALCL might be linked to the over replication and a higher mtDNA copy number in ASD than in controls [31]. These conditions were observed in cells in response to oxidative stress [31]. mtDNA encodes some of the proteins of the complexes of the electron transfer system (ETS). Thus, an increase in mtDNA copy number possibly leads to an increased expression of ETS complexes, resulting in a higher respiratory ET capacity. This, however, needs further clarification via real-time PCR to investigate the level of expression of ETS genes encoded in the mtDNA.

mtD from mtDNA mutations could lead to ETS instability, electron leakage, and increased ROS production [32]. Higher OXPHOS- and ET capacities would result in increased production of H_2_O_2_ and other ROS-inducing oxidative stress. A high level of H_2_O_2_ production is frequently observed in ASD patients compared to controls [33]. Our results are in line with another study that reported a 40%–50% increase in maximal respiratory rates in lymphoblasts of ASD patients as compared to the non-autism relative [34]. However, clarification of this hypothesis needs to be done via sequencing of the mtDNA of both cell lines.

CIV is the terminal respiratory complex of the ETS, which converts O_2_ to H_2_O. Diminished activity of ETS complexes has been reported as a prevalent mitochondrial abnormality associated with ASD [4]. For example, lowered activities of CI, CIII, and CIV were found in ASD studies using muscle cells [35] and in the brain from post-mortem ASD patients [36,37]. However, CIV mitochondrial-dependent oxygen consumption was not different in peripheral blood lymphocytes from children with autism versus controls [33]. On the other hand, increased activity of CIV was detected in brain tissue [38,39]. Similarly, in the present study of human lymphoblastoid cell lines, the CIV activity was higher in ALCL compared to NALCL. This occurs to cope with the increase in the ET capacity of the NADH- and succinate pathways (CI and CII), which results in higher production and the transfer of electrons toward CIV.

MtMP reflects the electrical potential difference across the mitochondrial inner membrane, where transmembrane pH difference is the driving force for ATP synthesis. Alterations in mtMP are strongly linked to the control of electron transfer and ATP synthesis [21,33,34] and are involved in apoptosis and necrosis [40]. In our study, the mtMP in ALCL was higher in S-OXPHOS respiration as compared to NALCL. This is in agreement with previous studies, which found that the mtMP in ASD is higher compared to controls [28,41]. Theoretically, the higher the mtMP would reflect the higher the energy capacity of the mitochondrial inner membrane and the higher the synthesis of ATP [42]. However, a high electric field is energetically expensive to maintain; thus, ion leakage would occur to compromise the mtMP [42]. This is reflected by a higher S-LEAK value in ALCL compared to NALCL. Mitochondrial ROS production by the mitochondrial transport system is increased at high membrane potential [42,43,44]. Fluctuations of the mtMP between different respiratory states are larger in ALCL than in NALCL. Such fluctuations may have deleterious effects on cell physiology [42]. Thus, high mtMP is potentially harmful to mitochondria and consequently to the cell [45].

On another note, classic mtD biomarkers are not always accurate. The elevation of pyruvate levels does not always reflect lower ET capacity. It could be due to impaired pyruvate metabolism and TCA cycle inhibition. Thus, an enzyme marker (e.g., CIV) can be used as a diagnostic tool for ASD and other mitochondrial diseases. It is also worth noting that ASD is a spectrum disorder in which different effects and causes may account for the link between mtD and various observed symptoms. Our findings suggest an involvement of mitochondria, particularly the complexes in the electron transport chain, such as CI, CII, and CIV, in the pathogenesis of ASD, specifically in idiopathic autism. This is tightly linked to the production of ROS by mitochondria as the possible underlying cause of ASD as CI is the biggest contributor of ROS production under pathological conditions, and both respiration rate and mtMP are proportional to H_2_O_2_ production [39,41,42].

These initial findings lead us to further directions that might include the exploration of mitochondrial elements in a bigger picture, such as measurement of mitochondrial density and determination of mitochondrial copy number. Other mitochondrial markers (e.g., measurement of the activity of citrate synthase) should be further explored for a wider understanding of the involvement of mitochondrial function in idiopathic autism. Altogether, these results indicate the correlation between mitochondrial function abnormalities and ASD. Novel and extended approaches are required to improve the diagnosis of mitochondrial function in ASD and find better intervention strategies.

## 5. Conclusions

Many mitochondrial diseases and mtD are characterized by lower activities of respiratory complexes and mitochondrial respiratory capacities, whereas we observed an opposite pattern in agreement with a few other studies on ASD. Thus, linking mtD to ASD should be revisited, and instead of mitochondrial ‘dysfunction’, mitochondrial ‘dysregulation’ represents a more accurate term in the context of ASD pathogenesis, especially in the case of idiopathic autism. Changes in mitochondrial respiratory capacities can be caused by a modification of mitochondrial quality or mitochondrial density. Both mechanisms may be implicated in the increased OXPHOS- and ET capacities, CIV activity, and mtMP in the autism lymphoblastoid cell lines, and may be linked to ROS production and oxidative stress, which needs further clarification and an interesting point to explore. These bioenergetic characteristics imply a disruption of nutrient homeostasis as a potential cause of the pathological symptoms prevalent in idiopathic autism.

## 6. Limitations & Future Direction

This is a preliminary study that uses only one pair of LCL; ALCL versus NALCL, comparing the mitochondrial functions which are the CIV activity, respiration of intact and permeabilized cells, specifically in the ROUTINE, LEAK, OXPHOS and ETS respirations, and mtMP. This study provides an insight into the differences in mitochondrial function between cells derived from an autistic child compared to his apparently healthy sibling. The findings might provide an overview of a subset of ASD. The generalization on ASD as a whole, therefore, must be made cautiously.

Future studies will, therefore, need to consider the gene mutation on both cell lines specifically in the mutations in the mtDNA.

## Figures and Tables

**Table 1:** Substrates, uncoupler, and inhibitors used in respirometry protocol to induce different respiratory states [20,21].

Substrates	Function	Concentration
Digitonin (Dig)	Plasma membrane permeabilization	7.5 µg∙mL^−1^ (NALCL) 27.5 µg∙mL^−1^ (ALCL)
Pyruvate (P)	NADH-generating substrate (Substrates for CI)	5 mM
Glutamate (G)		10 mM
Malate (M)		2 mM
Succinate (S)	CII substrate	10 mM
Ascorbate (As)	Maintains TMPD in a reduced state	2 mM
N,N,N′,N′-tetramethyl-p-phenylenediamine dihydrochloride (TMPD)	Substrate for reducing cytochrome *c* (Substrate for CIV)	0.5 mM
Cytochrome *c* (*c*)	Mitochondrial outer membrane permeability test	10 µM
ADP (D)	Substrate of ANT, F_1_F_0_-ATPase (Substrate for CV)	2.5 mM
Carbonyl cyanide m-chlorophenyl hydrazone, CCCP (U)	Uncoupler, protonophore	0.5 µM steps
Rotenone (Rot)	CI inhibitor	0.5 µM
Malonate (Mna)	CII inhibitor	5 mM
Antimycin A (Ama)	CIII inhibitor	2.5 µM
Sodium azide (Azd)	CIV inhibitor	100 mM
Oligomycin (Omy)	ATP synthase inhibitor	2.5 µM
Safranin (Saf)	Fluorophore, dye for measuring mitochondrial membrane potential	2 µM

**Table 2:** Definitions of respiratory states [22,23,24].

States and Ratios	Definition and Rate
N-pathway (CI-linked pathway)	Respiration induced by the addition of NADH-generating substrates. Electrons are transferred from CI to CIII and then to CIV.
S-pathway (CII-linked pathway)	Respiration induced by the addition of succinate and Rot. CI is inhibited by Rot while electrons can only be generated by CII, transferred to CIII and then to CIV.
NS-pathway (CI- and CII-linked pathways)	Respiration induced by the addition of NADH-generating substrates and succinate without Rot. Combination of both pathways (usual pathway) whereby electrons move from both CI and CII, to CIII and then to CIV.
ROUTINE, *R*	ROUTINE respiration controlled by intrinsic energy demand. This represents energy demand under steady-state conditions.
LEAK, *L*	LEAK respiration caused by proton leak, proton slip, cation cycling, and electron leak. *L* is measured in the presence of reducing substrate(s) but absence of ADP or after enzymatic inhibition of the phosphorylation system by Omy.
OXPHOS, *P*	Respiration in the ADP-stimulated state of oxidative phosphorylation; OXPHOS capacity.
ET, *E*	Oxygen consumption in the non-coupled state at optimum uncoupler concentration, ET capacity.
ROX, *Rox*	Residual oxygen consumption, measured after inhibition of the ETS.
(*E* − *P*)/*E*	Relative *E − P* excess capacity, defines the limitation of OXPHOS capacity exerted by the phosphorylation system.
(*P* − *L*)/*P*	OXPHOS coupling efficiency, combining the effects of coupling and limitation by the phosphorylation system.

## Data Availability

The data presented in this study are available upon request from the corresponding author as the data is not available elsewhere.
